# Infection prevention behaviors and perceptions of nurses in a medical intensive care unit

**DOI:** 10.1017/ash.2025.10231

**Published:** 2025-11-24

**Authors:** Frank A. Drews, Jeanmarie Mayer, Molly Leecaster, Lindsay Visonvsky, Tavis Huber, Matthew H. Samore

**Affiliations:** 1 Department of Psychology, University of Utah, Salt Lake City, UT, USA; 2 Division of Epidemiology, University of Utah Spencer Fox Eccles School Medicine, Salt Lake City, UT, USA

## Abstract

**Objective:**

During the severe acute respiratory syndrome coronavirus 2 (SARS-CoV-2) pandemic, recommended infection prevention practices for preventing transmission in acute healthcare settings included, at a minimum, universal source control with masking and eye protection within six feet of others and using a respirator when caring for individuals with COVID-19.

**Methods:**

A mixed methods study consisting of observations and self-reported infection prevention behaviors among critical care nurses identified high adherence to mask use in a medical intensive care unit (ICU) during the COVID-19 pandemic.

**Results:**

Surveyed nurses reported more barriers to proper use eye protection than with masking. We observed nurses wearing required eye-protection only 20% of the time when within 6 feet of others. Use of eye protection increased in the presence of patients while decreasing near other healthcare workers. In self-reported assessments, these nurses described decreased protective behaviors at work and at home after vaccination for COVID-19. However, self-reported masking in public remained high and was not impacted by vaccination status. Finally, nurses most frequently perceived high transmission risk of SARS-CoV-2 in the community and patient care settings, with lowest risk from co-workers and household members.

**Conclusions:**

Perceived risks of exposure to SARS-CoV-2 likely impact infection prevention behaviors. Differences in perceived risk between patient and peer groups provide insight into strategies for improving infection prevention behaviors in both pandemic and endemic multidrug-resistant organism settings.

The severe acute respiratory syndrome coronavirus 2 (SARS-CoV-2) is highly infectious, and early in the COVID-19 pandemic, was associated with significant morbidity and mortality. Initially, SARS-CoV-2 was believed to be transmitted primarily through droplet particles but is now recognized as transmitted mostly via short-range aerosols.^1–3^ Organisms transmitted via aerosols are challenging to prevent exposure for as they require higher levels of protective equipment; additionally, SARS-CoV-2-infected individuals shed virus prior to symptom onset and often remain asymptomatic.

Traditional healthcare infection prevention efforts primarily focus on preventing transmission between patient and healthcare worker (HCW) but with SARS-CoV-2, HCWs also faced risk of exposure from other HCWs. To protect HCWs, the Centers for Disease Control (CDC) provided enhanced infection prevention and control recommendations for healthcare settings, including universal masking and physical distancing.^3^

The implementation of protective measures may be uneven if individuals perceive different risk levels for acquiring SARS-CoV-2 from different groups. For example, HCWs may perceive transmission risk from other HCWs (familiar colleagues) as lower than from unfamiliar patients or community members.^4^ If so, HCW adherence to protocols intended to reduce transmission risk may differ between patient versus peer interactions. Additionally, public health efforts could affect perceived risk for certain interactions, such as risk of acquiring the virus from peers following an employee COVID-19 vaccination campaign.

The goal of this study was to document infection prevention behaviors of nurses on a clinical unit during the COVID-19 pandemic. We contextualized these behaviors by including HCWs’ risk perception for acquiring SARS-CoV-2, consistent with implications of what is referred to as the “behavioral immune system.”^5^

## Methods

This IRB approved study was conducted between February 24 and June 3, 2021, in the medical intensive care unit (MICU) of an academic medical center. The study consisted of direct observations of nurses’ infection prevention behaviors and survey responses from these nurses of their risk perceptions for COVID-19 and self-reported infection prevention measures. All MICU nurses were eligible to participate. Recruitment used a convenience sample by approaching nurses for permission to observe their activities while working. Participation was voluntary; nurses who consented to observation were also asked to complete a survey. All data was de-identified. At the time of the study, there were no outbreaks among patients or staff in the unit.

**Figure 1.**
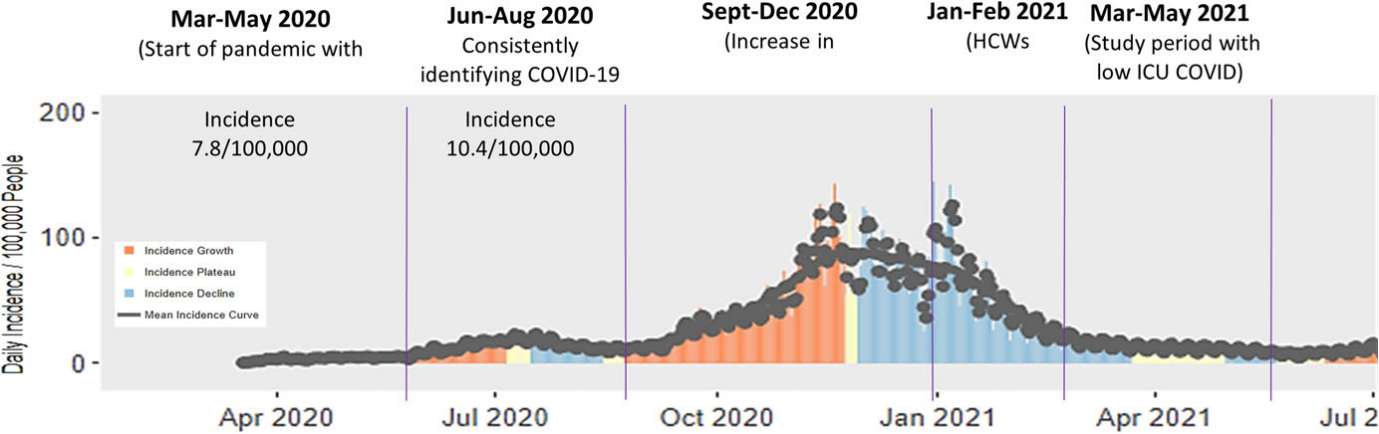
Utah-wide daily community COVID-19 incidence rates over varying periods. Study observations of nurses took place March through May 2021, and nurses were retrospectively asked about risk perception over varying time periods.

Context for the pandemic time line in Utah is provided in Figure 1 and shows the evolution in newly reported case rate using positive tests for SARS-CoV-2 from the start of the pandemic through the end of the study period. The average reported case rate of COVID-19 in the community during the study period was 8.0 per 100,000 people, which was considered low per CDC criteria.^6^ Over the study period, there were 1 to 4 new daily hospital admissions for COVID-19, which was lower than peak activity four months earlier (6–14 daily admissions for SARS-CoV-2). In addition, over 80% of the MICU HCWs were recently fully vaccinated by the start of the study through a COVID-19 vaccination campaign conducted mid-December 2020 through January 2021.

Among the community preventive measures in place during the first half of the study period were statewide physical distancing and mask mandates, both of which were lifted during the second half. Throughout the entire study period, K–12 schools held in-person classes with required masking. *Definitions of SARS-CoV-2 Transmission Risk Moments* To conceptualize transmission prevention behaviors, we augmented the World Health Organization’s (WHO) “5 Moments” framework which focuses on contact-based transmission routes to also include respiratory-based transmission routes and potential transmission from other HCWs.^7^ The WHO’s “patient zone” comprises the patient and the surrounding environmental surfaces potentially touched by the patient or HCWs. For this study, the “SARS-CoV-2 patient zone” included the air inside a patient room in airborne precautions. As transmission of SARS-CoV-2 may occur from HCW to HCW, we defined a “SARS-CoV-2-person zone” to include any other HCWs. The “SARS-CoV-2-person zone” was defined as unprotected contact for a potential transmission of SARS-CoV-2 (eg, not wearing a mask/protective eyewear within 6-feet from another person).

For this study, HCW infection prevention behaviors within a “SARS-CoV-2-person zone” were observed. If a HCW did not adhere to the healthcare system’s policies of wearing the required personal protective equipment (PPE) while in the “patient” and “person” zones, it was considered a potential transmission risk event.

### Observations

A trained observer shadowed nurses during their shift to conduct structured observations. Observations occurred Monday through Friday between 7am–4 pm. Standardized data were entered on an electronic tablet using coding software.^8^ Behaviors that included mask and glove wearing, and interaction with other HCWs were time-stamped to measure duration and frequency. Other behaviors (eg, hand hygiene, mask touching, inappropriate mask storage, and eating or drinking in clinical locations) were coded only as present/absent. Interactions were coded when individuals were within 6 feet of each other.

Four 1–2-hour pilot observations allowed for refinement of behavioral categories, tool usability, and establishing of an a priori inter-rater reliability of at least Cohen’s kappa of 0.85.

Two observers were trained to perform HCW observations by coding video recordings showing HCWs performing a range of tasks with varying levels of PPE adherence. The observers were given feedback on coding discrepancies compared to that of an expert’s. Observers then performed three 30-min observations of nurses in the MICU. Interrater reliability was determined as Cohen’s kappa of 0.91.

### Survey

At the conclusion of an observation nurses completed a 5-min survey. The survey included questions about perceived risk of SARS-CoV-2 transmission from patients, coworkers, family and community members (rank ordered), and about infection prevention practice barriers (7-point Likert-type scale). In addition, participants answered questions about their COVID-19 protective behaviors (eg, physical distancing, masking) for four time periods including initial recognition of COVID-19 with rare cases (March–May 2020); early in the pandemic with consistent reporting of infections (June–August 2020); as infection rates and hospitalizations peaked (September–December 2020); and when high COVID-19 rates were declining (January–February 2021).

*Definition of PPE Adherence* Nurses’ coded PPE use allowed assessment of adherence based on PPE requirements for universal masking, or as required by signage outside of a patient’s room. Lack of signage indicated absence of additional precautions other than universal masking (minimum = procedural mask and eye protection (i.e., unit-provided goggles)). Gloves and gown were required on entry to a room placed in contact precautions and for direct patient care in rooms with droplet precautions. For patients in droplet precautions, additional high-level respiratory protection with an N95/PAPR was required during aerosol generating procedures (AGP). Contact precautions signage in addition to airborne precautions, meant the patient had suspected or confirmed COVID-19.

### Statistical analysis

For the observational data counts and frequencies of observed behaviors were calculated overall and stratified by location and patient precautions status. Hand hygiene adherence at the point of potential transmission events was calculated by location. Adherence to PPE use during potential transmission risk moments was reported for each PPE item. We performed a logistic regression to examine the relationship between eye protection use and the location and presence of other HCWs. Survey data were analyzed by calculating the means and percentages of answers and t-tests comparing risk rankings. Statistical analyses were performed using R version 4.1.2.^9^

## Results

41 nurses participated in the study (100% participation rate).

### Observations of HCW infection prevention practices

The 41 observation sessions lasted 2,276 min (average duration: 56 min). We observed nurses’ interactions in patient rooms (926 min; 41% of total observation time), the hallway (629 min; 28%), the nursing station (521 min; 23%), and staff computer workstations (located just outside each patient room, 69 min; 3%) (see Table 1). In 31% of observations, nurses visited patient rooms with respiratory precautions.

**Table 1.** Summary of observation time, number of potential transmission events, and proportion of time personal protective equipment was used by HCW when in different general location of the HCW. The observed use of PPE by HCW when in patient rooms by precautions is also provided

Location of HCW	Duration (min)	Hand hygiene Events (no.)	Eat/Drink events (no.)	Touch events (no.)	Duration eye protection (%)	Duration gown (%)	Duration gloves (%)	Duration mask/PAPR (%)
Break room	16	1	0	0	0%	0%	0%	100%
Conference room	18	0	0	1	0%	0%	0%	100%
Hallway	602	223	4	15	19%	7%	9%	100%
Nurse’s station	521	21	6	20	19%	9%	5%	99%
Local work stations	69	7	1	4	8%	0%	19%	99%
Supply room	77	2	0	1	16%	5%	2%	100%
Other	47	1	0	0	1%	30%	7%	100%
Patient room	926	18	0	3	50%	42%	46%	100%
Observations in patient rooms by precaution categories								
Precautions	Duration (min)	Visit (no.)						
None	411	100	0	2	19%	10%	14%	100%
Contact	88	426	0	1	21%	33%	33%	100%
Respiratory*	426	50	0	0	86%	75%	80%	100%

* Respiratory includes (1) airborne plus contact, or (2) droplet. All respiratory precaution rooms required use of a mask with eye protection or PAPR. Droplet precautions instructed use gown and gloves with patient contact.

We observed high, but imperfect hand hygiene adherence during potential transmission events (81.6%; *n* = 223) in the hallway before entry or after exiting patient rooms. Adherence to hand hygiene was lower when nurses were inside patient rooms providing patient care (6.6%, *n* = 18) or at the nurse’s station (7.6%, *n* = 21). Consumption of food or beverages was only permitted in the break room, and nurses were only infrequently observed eating or drinking at the nurse’s station (*n* = 6) and in the hallway (*n* = 4) (Table 1). HCW face or mask touch potential contamination occurred at a rate of 1.15 times/hour and varied by location: 3.5 times/hour at staff workstations, 2.3 times/hour at the nurse’s station, and 1.5 times/hour in the hallway. Nurses rarely touched their faces or mask in patient rooms (0.2 times/hour).

We observed extremely high adherence in mask use among nurses in all unit locations and during all interactions in patient rooms (Table 1). Use of universal eye protection varied based on location; nurses followed policy 50% of the time in the patient room, 19% in the hallway and at the nurse’s station. However, nurses never wore eye protection in the break and conference rooms. The use of eye protection was low among contact-only or no-precautions patient rooms compared to 86% adherence in rooms in respiratory precautions. Despite the requirement to wear a gown and gloves on room entry for contact precautions, nurses were observed to adhere only a third of the time.

Table 2 summarizes the proportion of time nurses were observed wearing eye protection by location and presence of other HCWs. Nurses wore eye protection more often when in patient rooms but less often when around other HCWs. We investigated this finding in a multivariable logistic regression model and the interaction term between location and HCW was not statistically significant resulting in its removal from the model. The final model (Table 3) suggested that adherence to eye protection was significantly lower in all locations compared to the patient room (*p*-values < 0.0001), and also lower when in the presence of other HCWs (*p*-value < 0.0001).

**Table 2.** Adherence to eye protection by location and when with other HCWs

	Total time observed (min)		Duration (min) and percentages (%) with Eye protection	
Location	Alone	With other HCW	Alone	With other HCW
Patient room	565.5	369.1	351.0 (62.1)	113.8 (30.8)
Nurses station	347.8	173.5	78.6 (22.6)	21.6 (12.4)
Hallway	382.7	219.1	89.3 (23.3)	22.7 (10.4)
Other	155.9	71.4	9.3 (6.0)	8.9 (12.5)

**Table 3.** Logistic regression model testing use of eye protection by location or HCW-interaction

Model term	Odds ratio	95% confidence interval
Duration	0.96	0.93–0.99
With other HCW (vs not in presence of other HCW)	0.53	0.32–0.85
Hallway (vs Patient Room)	0.57	0.31–1.04
Nurses Station (vs Patient Room)	0.40	0.21–0.76
Other Locations (vs Patient Room)	0.27	0.14–0.54

### Survey

#### Barriers to adherence

**Figure 2.**
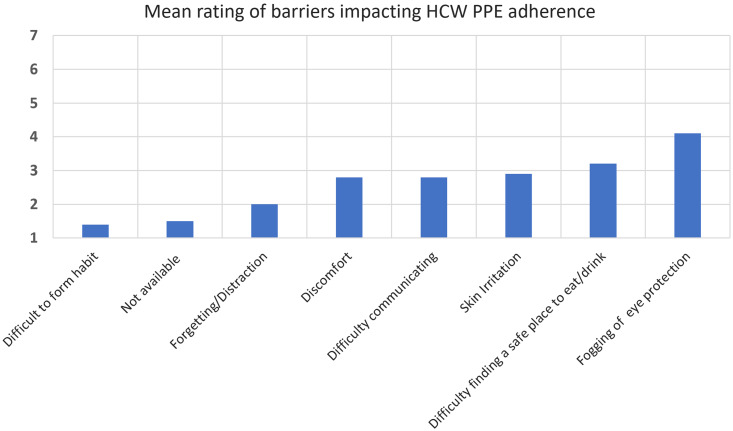
Barriers to adhering to universal masking, eye protection and related infection prevention practices using a Likert scale (1 = not an issue to 7 = extremely difficult).

Hospital policy required HCWs to wear a mask and eye protection at all times when around others in the unit. Participants ranked the impact of potential barriers to universal precautions on a 7-point Likert-type scale (from 1 (not an issue/no impact) to 7 (extremely difficult highest impact); Figure 2). Overall, nurses reported fogging of eye protection with the highest impact at 4.1, which may explain the low observed adherence. Despite complaints that masks caused skin irritation, discomfort, and difficulty communicating (rated respectively at 2.9, 2.8, and 2.8), these barriers did not affect adherence. In addition, while nurses rated difficulty in finding a safe place to eat or drink, they were near perfect in keeping a mask on when observed within 6 feet of other HCWs. Interestingly, other aspects like habit formation, lack of available PPE, and forgetting/distraction ranked low as barriers.

#### Risk perceptions and behaviors

Participants rank ordered their perceived risk of exposure to SARS-CoV-2 from four different social groups, with 4 as the highest and 1 as lowest risk. The mean rankings show that nurses perceived the lowest risk from their co-workers (mean = 1.1) or family members (mean = 1.2), compared to patients (mean = 1.7) or the community (mean = 2.0). There were statistically significant differences in average perceived risk levels between co-workers and community (t(36) = 3.1, *P* = 0.003) and co-workers and patients (t(36) = 2.7, *P* = 0.01).

**Figure 3.**
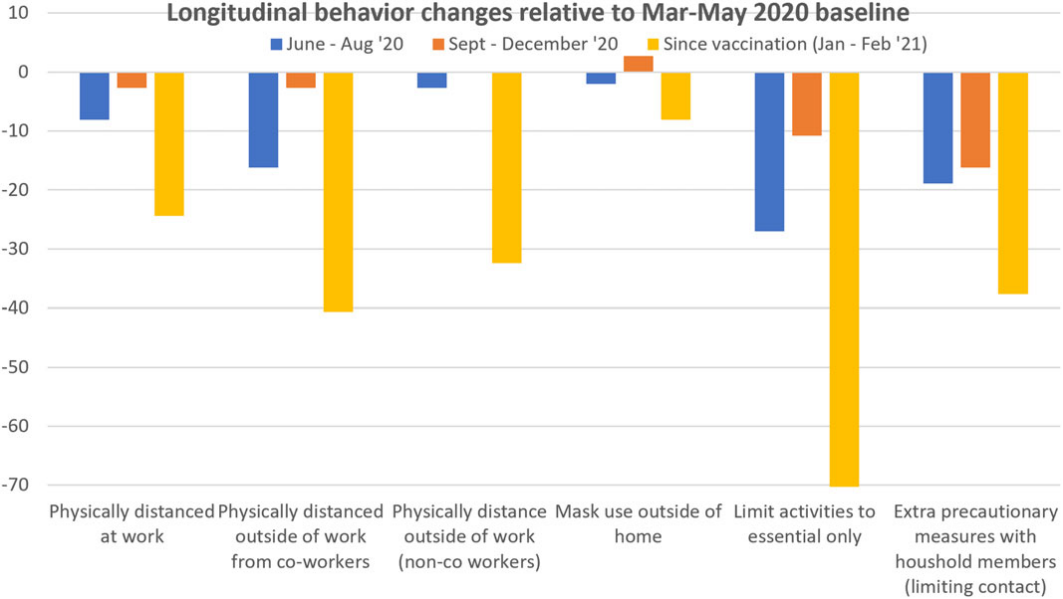
Changes in reported COVID-19 protective behaviors over time relative to baseline.

Participants were asked about their protective behaviors over four time periods (see Figure 1). Table 4 shows the percentage of nurses who reported use of protective behaviors at each time period. Figure 3 shows the relative changes in HCW protective behaviors from the initial baseline period. Not surprisingly, recent HCW vaccination along with declining hospitalized COVID-19 cases were concurrent with the largest behavioral changes: The reversal in limiting behaviors outside the home to essential activities is the most substantial, although outside of home mask use was largely unchanged.

**Table 4.** Percentage agreement to engaging in the described behaviors

	March–May 2020	June–August 2020	September–December 2020	January–February 2021*
Physically distanced at work	67.6%	59.5%	64.9%	43.2%
Physically distanced outside of work from co-workers	83.8%	67.6%	81.1%	43.2%
Physically distance from outside of work (non co-workers)	97.3%	94.6%	97.3%	64.9%
Mask use outside of work or home	83.8%	81.8%	86.5%	75.7%
Limit outside activities to essential only	86.5%	59.5%	75.7%	16.2%
Extra precautionary measures with household members (limiting contact)	70.3%	51.4%	54.1%	32.6%

*Majority of MICU nurses were fully vaccinated with 2 COVID-19 mRNA vaccines by mid Jan 2021.

## Discussion

Along with near perfect masking at the workplace, nurses reported continued masking in the community without substantial change over time, supporting the idea that participants believed in the effectiveness of masks and accepted universal masking at work as well as mask use in public.

However, use of eye protection was merely 20%. Nurses infrequently wore eye protection when caring for patients who were not in respiratory precautions and at best, they donned only 50% of the time in higher risk settings. This suggests that HCWs may have believed that protective eye equipment was not important in reducing their risk of exposure to SARS-CoV-2, or not enough of a benefit to outweigh the perceived negative impact of eyewear. An even more nuanced picture emerges when adherence to eye protection was examined by location and presence of other HCWs. Nurses had the highest compliance of wearing eye protection while in patient rooms and when alone. Since adherence to eye protection differed when nurses were inside versus outside of patient rooms and when around other HCWs, there are likely additional influences mitigating behavior. Nurses were also observed to contaminate their face/masks relatively frequently at the nursing station and hallway, but rarely in patient rooms. One interpretation is that nurses perceived patient care as a higher risk exposure than from co-workers.

When analyzing infection risk perceptions, nurses ranked infection risk from other HCWs and household members lower compared to risk associated with patients and the general community. This difference is reflected in the observational data, as adherence to infection prevention measures was high during patient encounters and lower when in the presence of co-workers. With the majority of HCWs being vaccinated at the time of the study, there is reason to believe that the infection risk was relatively low, however, household members were unlikely to be vaccinated, suggesting an underestimation of risk consistent with the idea of underestimation of in-group risk.^4,5^

Other factors may have influenced low adherence to PPE, including the comfort and usability of protective eye gear and inconvenience of gown donning/doffing. The most significant barrier to wearing PPE was fogging of the protective eye equipment. Addressing inconvenience, discomfort, or poor usability of PPE would likely improve HCW adherence and safety.^10^

HCWs likely became more “comfortable” with SARS-COV-2 over time, as increasing numbers of individuals in the community, including nurses, acquired partial immunity through natural infection and by vaccination. Our observations of nurses’ infection prevention occurred at a time in the pandemic when COVID-19 rates were on the decline and HCW had just recently been fully vaccinated. When SARS-CoV-2 was a newly recognized pathogen, hand hygiene rates in the MICU rose from the previous year of 87% to a high of 97%. As more focus and HCW effort shifted to respiratory protection, hand hygiene rates fell to the previous baseline of 86%. At the time of the study hand hygiene decreased to a low of 81%, and survey results indicate lower adherence to physical distancing with other HCWs and return to normal outside activities.

Overall, this study suggests that the nurses’ infection prevention practices were influenced by their risk perception which evolved over the pandemic time line. The results are consistent with the idea of psychological factors affecting infection risk perceptions and infection prevention behavior. Most importantly, these factors can lead to biases that may not reflect objective risks. Infection prevention and healthcare safety programs can learn from these findings beyond the pandemic’s exceptional circumstances. Successful implementation of infection prevention programs may benefit from inclusion of risk messaging strategies that accurately convey infection risks. Such messaging should also address psychological biases that result in inaccurate risk perceptions with direct impact on behavior.

## Limitations

Observations in this study were limited to weekdays and the day shift. Infection prevention behavior at other times may have differed from our reported data, possibly overestimating adherence. While on average each observation period was substantial, the number of observation sessions and nurses observed is relatively small. Since participation was voluntary and only nurses were recruited, there may have been self-selection, and our results may not be generalizable to infection prevention practices of a broader HCW population.
