# Infection prevention behaviors and perceptions of nurses in a medical intensive care unit – CORRIGENDUM

**DOI:** 10.1017/ash.2026.10301

**Published:** 2026-01-26

**Authors:** Frank A. Drews, Jeanmarie Mayer, Molly Leecaster, Lindsay Visnovsky, Tavis Huber, Matthew H. Samore

In the initial publication of this article,^1^ author Lindsay Visnovsky’s surname was misspelled. The article has been updated to correct the spelling of the name.
